# A systematic analysis of gut microbiome characteristics in Chinese patients with gestational diabetes mellitus

**DOI:** 10.3389/fnut.2026.1902356

**Published:** 2026-09-22

**Authors:** Yukun Wang, Hui Zheng, Chunyi Yue, Jinyan Liang, Xinyu He, Lei Mo, Jian Huang, Xiangyuan Yu

**Affiliations:** 1 Guangxi Key Laboratory of Diabetic Systems Medicine, Guilin Medical University, Guilin, China; 2 Department of Epidemiology and Health Statistics, School of Public Health, Guilin Medical University, Guilin, China; 3 Department of Obstetrics and Gynecology, The Second Affiliated Hospital of Guilin Medical University, Guilin, China; 4 Department of Cell and Genetics, School of Basic Medical Sciences, Guilin Medical University, Guilin, China

**Keywords:** association, characteristics, gestational diabetes mellitus, gut microbiota, systematic review

## Abstract

**Objective:**

To systematically analyze the change pattern of core gut microbiota in Chinese gestational diabetes mellitus (GDM) patients and provide basis for GDM early microecological screening and probiotic intervention.

**Methods:**

Databases including PubMed, web of science, CNKI, and Wanfang were searched to identify reports on the association between gut microbiota and GDM. A quantitative system evaluation was conducted to assess the changes in core gut microbiota levels (such as *Bifidobacterium, Lactobacillus*, etc.) in GDM.

**Results:**

Totally, 31 studies involving 6,157 subjects were included. Systematic analysis revealed that levels of *Bifidobacterium* (SMD = −2.47, 95%*CI*: −2.86 to −2.09) and *Lactobacillus* (SMD = −1.82, 95% CI: −2.04 to −1.60) in the GDM were significantly lower than those in controls; while *Enterobacteriaceae* (SMD = 1.75, 95% CI: 1.53 to 1.98) and *Enterococcus* (SMD = 1.18, 95%CI: 1.03 to 1.32) were significantly higher than those in non-GDM. Sensitivity analysis shows that the above findings are relatively robust.

**Conclusion:**

GDM exhibit significant gut microbiota dysbiosis. The reduction in *Bifidobacterium* and *Lactobacillus* and the increase in *Enterobacteriaceae* and *Enterococcus* may be important micro biological features for the onset and progression of GDM.

## 1 Introduction

Gestational diabetes mellitus (GDM) refers to abnormalities in glucose metabolism of varying degrees that first occur or are diagnosed during pregnancy. In recent years, with the change of lifestyle and the postponement of childbearing age, the incidence rate of GDM continues to rise globally. In China, it has reached 14.8%∼17.8%, which is far higher than the global average incidence rate. It has become one of the most common complications during pregnancy, which seriously threatens the health of mothers and infants (1, 2). GDM not only increases the risk of preeclampsia (PE), cesarean delivery, and postpartum T2DM in pregnant women, but may also lead to adverse pregnancy outcomes such as fetal malformations, fetal growth restriction, and has a profound impact on long-term metabolic health of the offspring (3, 4).

The gut microbiota, often referred to as the body’s “forgotten organ,” plays a crucial role in maintaining host metabolic homeostasis and immune regulation (5). Studies have demonstrated that gut dysbiosis is closely associated with the insulin resistance, chronic low-grade inflammation, and metabolic diseases occurrence (6). During pregnancy, significant changes in gut microbiota occur due to changes in hormone levels and physiological insulin resistance. Growing evidence indicates that the gut microbiota composition in women with GDM differs from that in healthy pregnant women, characterized by a reduction in beneficial bacteria (such as Bifidobacterium and Lactobacillus) and an increase in opportunistic pathogens (such as Enterobacter and Enterococcus) (7). For example, metabolites such as short chain fatty acids and bacteriocins produced by *Bifidobacterium* and *Lactobacillus* are considered to have potential anti-inflammatory and antioxidant effects, thereby improving the intestinal microenvironment and metabolic status (8). As opportunistic pathogens, *Enterobacteriacea*e and *Enterococcus* proliferate excessively when the microbiota is disrupted, producing endotoxins such as lipopolysaccharides to activate inflammatory pathways, thereby promoting inflammatory responses and insulin resistance (9).

Accumulating evidence has highlighted the association between GDM and the gut microbiota in Chinese populations, offering critical insights into the distinct microbial signatures of this ethnic group. However, substantial heterogeneity in study design particularly regarding sample selection, detection methodologies, and outcome measures, has yielded inconsistent findings. Consequently, we aim to conduct a systematic analysis to delineate the core gut microbiota characteristics of GDM in Chinese women, thereby providing robust evidence for early screening, prevention, and probiotic interventions.

## 2 Materials and methods

### 2.1 Inclusion and exclusion criteria

Studies were included if they met the following PICOS criteria: (1) Population: Chinese pregnant women, comprising cases with GDM and controls with normal glucose tolerance (NGT); (2) Study Design: Case-control or cohort studies; (3) Outcome Measures: Quantitative data on gut microbiota composition were explicitly reported. Specifically, data for at least one of the following taxa were required: *Bifidobacterium*, *Lactobacillus*, *Enterobacteriaceae*, *Enterococcus*, *Bacteroides*, or *Fusobacterium*.

Studies were excluded based on the following criteria: (1) Data Integrity and Redundancy: Duplicate publications, with overlapping cohorts, or retracted articles; (2) Publication Type: Reviews, systematic reviews, conference abstracts, case reports, and animal studies; (3) Population Specificity: Non-Chinese populations; pre-existing diabetes mellitus; antibiotic or probiotic use within 4 weeks prior to sampling; comorbidities such as severe gastrointestinal or autoimmune diseases; and multiple gestations; (4) Methodological Flaws: Incomplete data reporting or inconsistent outcome measures that precluded meta-analysis.

### 2.2 Search strategy

A comprehensive literature search was performed across eight databases, including PubMed, and Web of Science, CNKI, CBM, Wanfang, VIP, Chaoxing, covering the period from their inception to January 2026. The search strategy combined MeSH terms and free-text words related to “gestational diabetes mellitus” and “gut microbiota” (including synonyms). Furthermore, manual retrieval of academic compilations, dissertations, and grey literature was conducted to minimize publication bias. The final selection of studies was based on predefined inclusion criteria after merging and deduplicating search results.

### 2.3 Literature screening and data extraction

Two reviewers independently screened all records against the predefined inclusion and exclusion criteria. The initial screening was performed by examining titles and abstracts to exclude irrelevant studies, followed by a full-text review to determine final eligibility. Data extraction was conducted in accordance with the objectives of this meta-analysis. Extracted information included study characteristics (e.g., author, publication year, region), sample size, gestational age at sampling, pre-pregnancy BMI, parity, and the specific methods and quantitative data pertaining to target gut microbiota profiling.

### 2.4 Assessment of risk of bias in included studies

The Newcastle-Ottawa Scale (NOS) was used for quality assessment (10). Quality was evaluated based on study population selection, comparability of groups, and measurement of exposure factors. The maximum score is 9 points; a score of ≥ 7 was considered high-quality research, while a score < 3 was considered low-quality literature. All studies included were high-quality studies with a score ≥ 7.

### 2.5 Statistical analysis

Meta-analyses were conducted using Stata version 12.0. For continuous variables, standardized mean differences (SMDs) with corresponding 95% confidence intervals (CIs) were calculated. Statistical heterogeneity was quantified using the statistic. A fixed-effects model was applied when I^2^≤ 50% and *P* > 0.05; conversely, a random-effects model was employed for I^2^> 50% and *P* ≤ 0.05. In the presence of significant heterogeneity, subgroup analyses were performed based on detection methods, pre-pregnancy BMI, and gestational age to explore potential sources of variation. Sensitivity analyses were also conducted by sequentially omitting individual studies to validate the stability of the pooled estimates. Publication bias was evaluated using Begg’s test.

## 3 Results

### 3.1 Literature search and basic characteristics

The initial search identified 1,612 records. After removing 809 duplicates, 803 records remained for title and abstract screening. Following the exclusion of systematic reviews, conference abstracts, animal studies, non-Chinese populations, and irrelevant topics, 151 articles were retained for full-text review. Subsequently, 120 studies were excluded for the following reasons: duplicate publication or overlapping data (*n* = 12); incomplete data or mismatched outcome measures (*n* = 101), defined as studies reporting only alpha/beta diversity indices without quantitative data for six pre-specified genera, studies focusing on gut mycobiome or metabolomic profiles rather than bacterial composition, studies with data aggregated at phylum or family levels that could not be extracted to genus level, or studies lacking means and standard deviations required for effect size calculation; inability to retrieve the full text (*n* = 1); formal retraction (*n* = 1); and confounding factors or baseline discrepancies (*n* = 5), primarily involving significant differences between case and control groups at baseline in maternal age, pre-pregnancy BMI, or comorbidities such as hypertension, which are known to influence both gut microbiota composition and GDM risk. Ultimately, 31 case-control studies (11–41) were included, encompassing 6,157 participants (2,964 with GDM and 3,193 controls). See Figure 1 and Tables 1, 2. This systematic review and meta-analysis was conducted in accordance with the PRISMA reporting guidelines.

**FIGURE 1:**
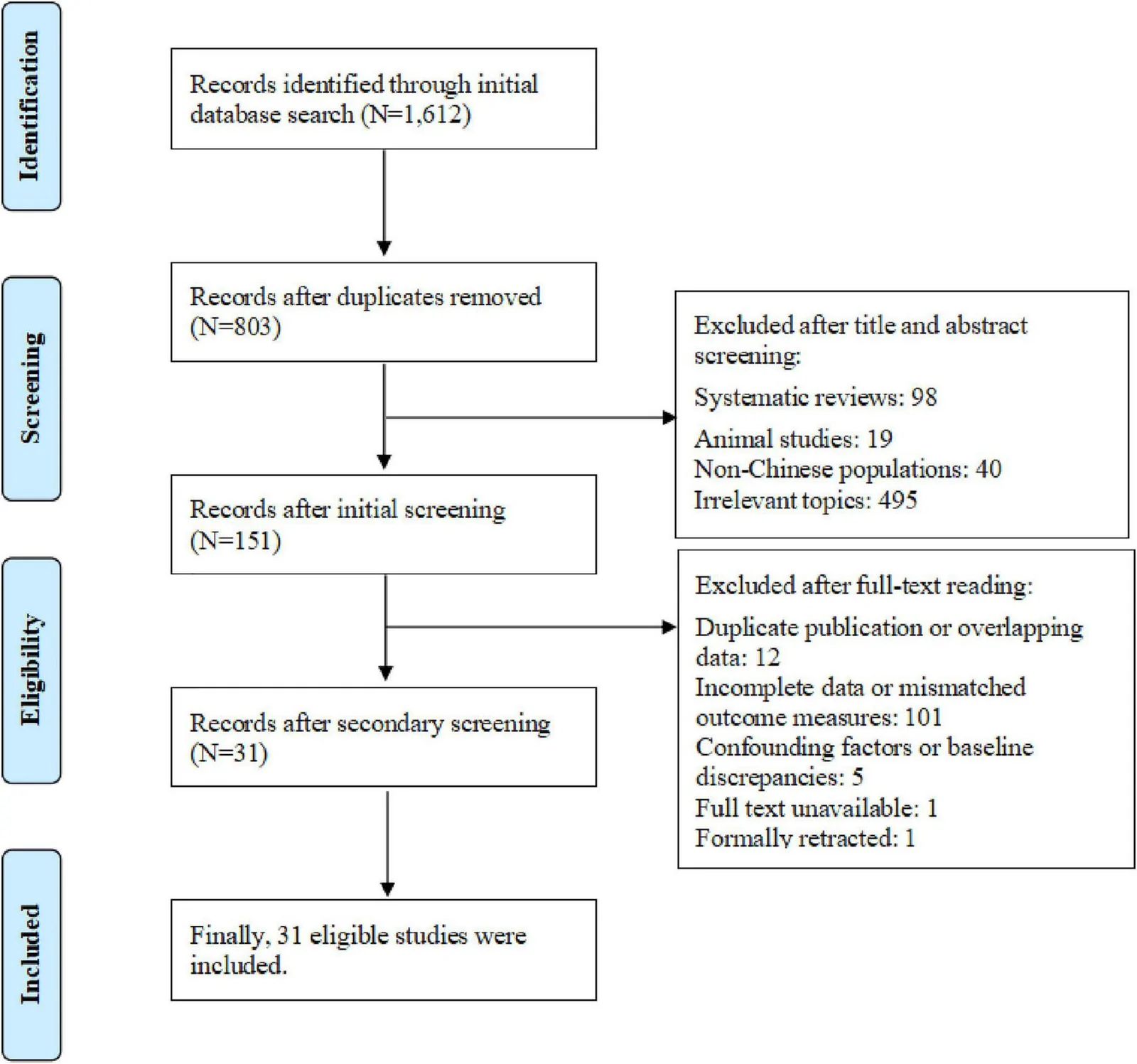
Literature screening flowchart.

**TABLE 1:** Basic characteristics of included studies.

Author (year)	Sample size (GDM/control)	Detection method	Age (GDM/control)	Pre-pregnancy BMI (GDM/control)	Gestational week (GDM/control)	Gravidity (GDM/control)	Outcome	NOS score
Chen et al. (11)	38/50	qPCR	27.81 ± 6.13/28.01 ± 6.05	–	25.37 ± 3.05/26.08 ± 3.44	1.32 ± 0.32/1.40 ± 0.29	abe	7
Chen (12)	50/50	qPCR	28.00 ± 8.00/27.00 ± 9.00	25.08 ± 2.93/24.65 ± 3.25	–	–	ab	7
Zhang et al. (13)	116/101	Culture counting	27.63 ± 4.80/25.71 ± 5.05	23.24 ± 2.30/22.43 ± 2.19	24.71 ± 2.14/25.57 ± 2.58	1.17 ± 0.34/1.11 ± 0.35	abcdef	7
Cao et al. (14)	111/111	Culture counting	29.60 ± 7.40/29.30 ± 7.00	22–24	–	–	abc	7
Wang et al. (15)	45/45	Culture counting	25.60 ± 2.58/26.04 ± 2.35	–	27.28 ± 5.06/26.80 ± 4.76	–	abce	7
Zhao et al. (16)	30/30	qPCR	24.10 ± 2.63/23.17 ± 2.59	–	25.17 ± 1.12/25.20 ± 1.16	–	a	7
Wu (17)	167/501	Culture counting	27.84 ± 2.16/28.20 ± 2.54	22.68 ± 3.20/22.40 ± 3.15	27.84 ± 2.16/28.20 ± 2.54	1.30 ± 0.41/1.27 ± 0.31	abedf	7
Liu et al. (18)	110/110	Culture counting	26.52 ± 3.90/25.82 ± 4.16	–	25.82 ± 3.25/25.34 ± 2.96	1.21 ± 0.23/1.29 ± 0.31	abcdef	7
Wang (19)	160/80	Culture counting	28.20 ± 1.70/ 28.20 ± 1.70	–	–	2.00 ± 0.20/2.00 ± 0.20	abcdf	7
Zhang et al. (20)	83/77	Culture counting	28.30 ± 2.40/28.30 ± 2.40	–	27.80 ± 4.90/27.80 ± 4.90	–	abce	7
Zhang et al. (21)	117/117	Culture counting	25.80 ± 2.30/25.90 ± 2.40	–	25.30 ± 2.20/25.50 ± 2.10	1.30 ± 0.20/1.30 ± 0.20	abcdef	7
Zhang et al. (22)	76/76	qPCR	29.57 ± 3.67/28.99 ± 3.57	21.35 ± 2.97/21.57 ± 3.12	26.38 ± 1.98/27.01 ± 2.03	–	ab	7
Chen et al. (23)	52/74	Culture counting	25.40 ± 1.80/25.10 ± 1.70	–	26.2 ± 1.3/25.9 ± 1.4	1.30 ± 0.30/1.30 ± 0.30	abcdef	7
Zeng et al. (24)	40/50	Culture counting	27.43 ± 5.81/27.89 ± 6.06	–	27.16 ± 4.24/27.74 ± 5.15	–	abcd	7
Huang et al. (25)	182/130	Culture counting	28.21 ± 2.55/27.84 ± 2.170	–	25.95 ± 1.91/26.04 ± 1.88	1.26 ± 0.31/1.31 ± 0.40	abcdf	7
Huang et al. (26)	40/80	Culture counting	27.85 ± 1.74/27.82 ± 1.77	–	26.32 ± 1.48/26.31 ± 1.47	2.06 ± 0.37/2.04 ± 0.38	abcde	8
Li (27)	56/56	qPCR	32.54 ± 3.87/32.74 ± 4.03	23.31 ± 2.25/23.54 ± 2.10	–	1.95 ± 0.40/2.01 ± 0.50	ab	7
Ma et al. (28)	95/80	qPCR	30.20 ± 2.10/29.70 ± 2.20	–	27.40 ± 3.60/27.60 ± 3.70	2.30 ± 0.70/2.60 ± 0.80	abce	7
Xiao et al. (29)	62/60	Culture counting	27.45 ± 5.79/27.91 ± 6.04	–	27.14 ± 4.22/27.76 ± 5.13	–	abcd	7
Yang et al. (30)	70/30	Culture counting	26.17 ± 3.03/27.01 ± 3.14	–	–	–	abedf	8
Yang et al. (31)	80/80	qPCR	27.63 ± 2.51/28.28 ± 2.37	–	25.04 ± 2.45/25.13 ± 2.51	−	abcdef	7
Zhang et al. (32)	100/80	qPCR	32.86 ± 2.30/32.90 ± 2.27	27.68 ± 2.21/27.70 ± 1.96	25.15 ± 2.07/24.99 ± 2.10	2.03 ± 0.50/2.05 ± 0.47	ab	7
Fang et al. (33)	108/108	Culture counting	27.62 ± 2.84/28.03 ± 1.78	–	–	2.30 ± 1.05/2.27 ± 1.10	abcdf	7
Guo et al. (34)	100/80	Culture counting	29.17 ± 5.82/29.11 ± 5.87	–	39.28 ± 0.94/39.23 ± 0.96	–	abcdf	7
Sun (35)	74/142	qPCR	27.50 ± 1.60/27.30 ± 1.80	–	–	–	ab	7
Yuan et al. (36)	88/120	Culture counting	30.98 ± 2.51/30.86 ± 2.43	–	25.88 ± 1.21/25.76 ± 1.15	–	abcdf	7
Zhang et al. (37)	256/253	qPCR	25.62 ± 1.74/25.33 ± 1.65	21.86 ± 2.83/21.45 ± 2.72	–	1.85 ± 0.38/1.91 ± 0.42	abcde	8
Wang (38)	142/142	qPCR	–	–	–	–	a	9
Huang et al. (39)	120/100	Culture counting	30.81 ± 2.46/30.79 ± 2.35	27.15 ± 3.22/26.79 ± 3.46	–	–	abcdf	7
Li et al. (40)	100/100	qPCR	30.28 ± 3.15/30.45 ± 3.12	–	25.45 ± 1.55/25.51 ± 1.58	2.45 ± 0.55/2.51 ± 0.58	abcdf	7
Ma et al. (41)	96/80	Culture counting	27.50 ± 3.56/26.50 ± 3.24	–		–	abde	7

a, *Bifidobacterium*; b, *Lactobacillus*; c, *Enterobacteriaceae*; d, *Enterococcus*; e. *Bacteroides*; f, *Fusobacterium*.

**TABLE 2:** Data of outcome measures of included studies.

Author (year)	*Bifidobacterium* (GDM/control)	*Lactobacillus* (GDM/control)	*Enterobacteriaceae* (GDM/control)	*Enterococcus* (GDM/control)	*Bacteroides* (GDM/control)	*Fusobacterium* (GDM/control)	Unit (CFU/copies)
Chen et al. (11)	7.04 ± 0.96/9.52 ± 0.85	7.12 ± 0.56/8.67 ± 0.72	–	–	8.43 ± 0.73/9.55 ± 0.76	–	lg copies/g
Chen (12)	10.77 ± 0.14/11.56 ± 0.18	5.34 ± 0.68/6.65 ± 0.96	–	–	–	–	lg copies/g
Zhang et al. (13)	7.35 ± 0.49/8.06 ± 0.32	5.31 ± 0.68/6.75 ± 1.86	9.54 ± 1.08/8.26 ± 0.90	8.30 ± 1.42/7.12 ± 1.80	9.45 ± 1.68/8.94 ± 1.43	3.35 ± 0.50/3.28 ± 0.91	lg CFU/g
Cao et al. (14)	6.3 ± 0.70/10.4 ± 0.80	5.20 ± 0.60/6.70 ± 0.80	8.90 ± 1.10/7.00 ± 0.90	–		–	lg CFU/g
Wang et al. (15)	7.04 ± 0.68/9.13 ± 0.85	6.76 ± 0.74/8.25 ± 0.80	10.76 ± 1.12/7.85 ± 0.83	–	7.45 ± 0.61/9.02 ± 0.78	–	lg CFU/g
Zhao et al. (16)	9.85 ± 1.08/10.81 ± 1.0	–	–	–			lg copies/g
Wu (17)	7.1 ± 0.58/8.10 ± 0.44	5.3 ± 0.79/6.87 ± 0.91	9.6 ± 0.98/8.1 ± 0.92	8.44 ± 1.12/7.03 ± 1.24		3.2 ± 0.67/3.22 ± 0.70	lg CFU/g
Liu et al. (18)	7.48 ± 0.52/8.17 ± 0.43	5.42 ± 0.79/6.86 ± 1.91	9.65 ± 0.97/8.37 ± 0.85	8.29 ± 1.53/7.23 ± 1.95	9.56 ± 1.79/8.83 ± 1.30	3.46 ± 0.61/3.39 ± 0.85	lg CFU/g
Wang (19)	7.08 ± 0.27/8.12 ± 0.38	5.32 ± 0.75/6.74 ± 0.84	9.58 ± 0.91/8.05 ± 0.83	8.41 ± 1.10/7.01 ± 1.14		3.19 ± 0.65/3.20 ± 0.68	lg CFU/g
Zhang et al. (20)	6.92 ± 0.71/9.21 ± 0.78	6.82 ± 0.88/8.31 ± 0.79	11.02 ± 1.02/8.01 ± 0.94	–	7.32 ± 0.59/8.99 ± 0.62	–	lg CFU/g
Zhang et al. (21)	7.52 ± 0.47/8.23 ± 0.51	5.36 ± 0.81/6.68 ± 1.79	9.72 ± 0.98/8.41 ± 0.79	8.33 ± 1.57/7.19 ± 1.88	9.68 ± 1.67/8.76 ± 1.48	3.51 ± 0.69/3.47 ± 0.65	lg CFU/g
Zhang et al. (22)	10.24 ± 2.38/11.54 ± 2.37	5.87 ± 1.39/6.59 ± 1.47	–	–	–	–	lg copies/g
Chen et al. (23)	7.39 ± 0.5/8.08 ± 0.35	5.38 ± 0.73/6.82 ± 0.9	9.63 ± 1.16/8.26 ± 0.91	8.39 ± 1.21/7.01 ± 0.79	9.69 ± 0.71/8.67 ± 0.61	3.37 ± 0.46/3.33 ± 1.00	lg CFU/g
Zeng et al. (24)	7.26 ± 0.58/9.91 ± 0.69	5.32 ± 0.55/7.09 ± 0.64	9.45 ± 1.15/8.14 ± 0.89	8.19 ± 1.27/7.18 ± 1.08	–	–	lg CFU/g
Huang et al. (25)	7.11 ± 0.59/8.11 ± 0.45	5.31 ± 0.8/6.88 ± 0.92	9.61 ± 0.99/8.11 ± 0.93	8.45 ± 1.13/7.04 ± 1.25	–	3.21 ± 0.68/3.23 ± 0.71	lg CFU/g
Huang et al. (26)	7.40 ± 0.53/8.05 ± 0.67	5.36 ± 0.71/6.81 ± 0.92	9.54 ± 1.15/8.24 ± 0.96	8.24 ± 1.23/7.05 ± 0.81	9.68 ± 0.77/8.83 ± 0.74		lg CFU/g
Li (27)	11.34 ± 0.21/12.95 ± 0.34	5.32 ± 0.14/6.53 ± 0.25	–	–	–	–	lg copies/g
Ma et al. (28)	6.98 ± 0.79/9.22 ± 0.84	6.82 ± 0.91/8.34 ± 0.8	11.09 ± 1.08/8.09 ± 1.03	–	7.34 ± 0.64/8.18 ± 0.69	–	lg copies/g
Xiao et al. (29)	7.28 ± 0.56/9.93 ± 0.67	5.34 ± 0.53/7.11 ± 0.62	9.47 ± 1.13/8.16 ± 0.87	8.21 ± 1.25/7.20 ± 1.06	–		lg CFU/g
Yang et al. (30)	7.34 ± 0.61/8.1 ± 0.45	5.32 ± 0.69/6.88 ± 0.87	9.64 ± 1.03/8.21 ± 0.97	8.34 ± 1.12/6.92 ± 0.77	–	3.23 ± 0.43/3.31 ± 0.37	lg CFU/g
Yang et al. (31)	7.39 ± 0.66/10.63 ± 0.73	5.39 ± 0.68/7.73 ± 1.43	9.77 ± 0.98/7.01 ± 0.63	8.3 ± 1.46/7.01 ± 0.92	9.47 ± 1.65/7.86 ± 1.42	3.47 ± 0.59/3.51 ± 0.72	lg copies/g
Zhang et al. (32)	5.12 ± 1.23/7.45 ± 1.18	5.29 ± 1.35/6.60 ± 1.09	–	–	–	–	lg copies/g
Fang et al. (33)	7.11 ± 0.35/8.25 ± 0.28	5.17 ± 0.84/6.85 ± 0.79	9.6 ± 0.82/8.14 ± 0.78	8.52 ± 1.09/7.03 ± 0.98	–	3.22 ± 0.69/3.18 ± 0.58	lg CFU/g
Guo et al. (34)	7.41 ± 0.52/8.09 ± 0.37	5.39 ± 0.74/6.82 ± 0.91	9.64 ± 1.14/8.32 ± 0.89	8.40 ± 1.23/7.02 ± 0.79	−	3.38 ± 0.46/3.34 ± 1.01	lg CFU/g
Sun (35)	6.96 ± 0.45/8.85 ± 0.5	7.03 ± 0.5/8.31 ± 0.61	10.03 ± 0.7/8.13 ± 0.72	8.51 ± 0.66/7.53 ± 0.61	9.21 ± 0.52/8.05 ± 0.46	3.65 ± 0.08/3.05 ± 0.06	lg copies/g
Yuan et al. (36)	7.24 ± 0.65/8.19 ± 0.49	5.22 ± 0.84/6.82 ± 0.97	9.78 ± 0.99/8.15 ± 0.96	8.56 ± 1.18/7.05 ± 1.28	−	3.22 ± 0.69/3.24 ± 0.76	lg CFU/g
Zhang et al. (37)	7.34 ± 0.38/8.13 ± 0.46	5.41 ± 0.72/6.75 ± 0.93	9.75 ± 1.23/8.32 ± 0.94	8.52 ± 1.19/7.15 ± 0.84	9.58 ± 0.75/8.46 ± 0.65	−	lg copies/g
Wang (38)	6.02 ± 0.9/6.25 ± 0.90	–	–	–	–		lg copies/g
Huang et al. (39)	7.22 ± 0.66/8.2 ± 0.46	5.19 ± 0.88/6.83 ± 0.99	9.69 ± 0.96/8.21 ± 0.89	8.45 ± 1.17/7.11 ± 1.22	−	3.19 ± 0.81/3.23 ± 0.69	lg CFU/g
Li et al. (40)	7.01 ± 0.63/8.09 ± 1.01	5.38 ± 0.63/6.05 ± 0.87	9.4 ± 1.33/8.32 ± 1.4	8.56 ± 1.12/6.52 ± 0.78	−	3.38 ± 0.50/3.35 ± 0.47	lg copies/g
Ma et al. (41)	6.88 ± 0.72/8.85 ± 0.9	7.12 ± 0.8/8.45 ± 0.92	−	8.55 ± 0.93/7.54 ± 0.88	9.25 ± 1.25/8.05 ± 0.85	−	lg CFU/g

### 3.2 Meta-analysis of associations between target gut microbiota and GDM

#### 3.2.1 Bifidobacterium and GDM

As illustrated in Figure 2A, the meta-analysis of 31 studies (11–41) (encompassing 2,964 GDM cases and 3,193 controls) demonstrated substantial heterogeneity regarding Bifidobacterium abundance (I^2^ = 97.10%, *P* < 0.001). Using a random-effects model, we observed that Bifidobacterium levels were significantly lower in the GDM group compared to controls (SMD = −2.47, 95%CI: −2.86 to −2.09, *P* < 0.001).

**FIGURE 2:**
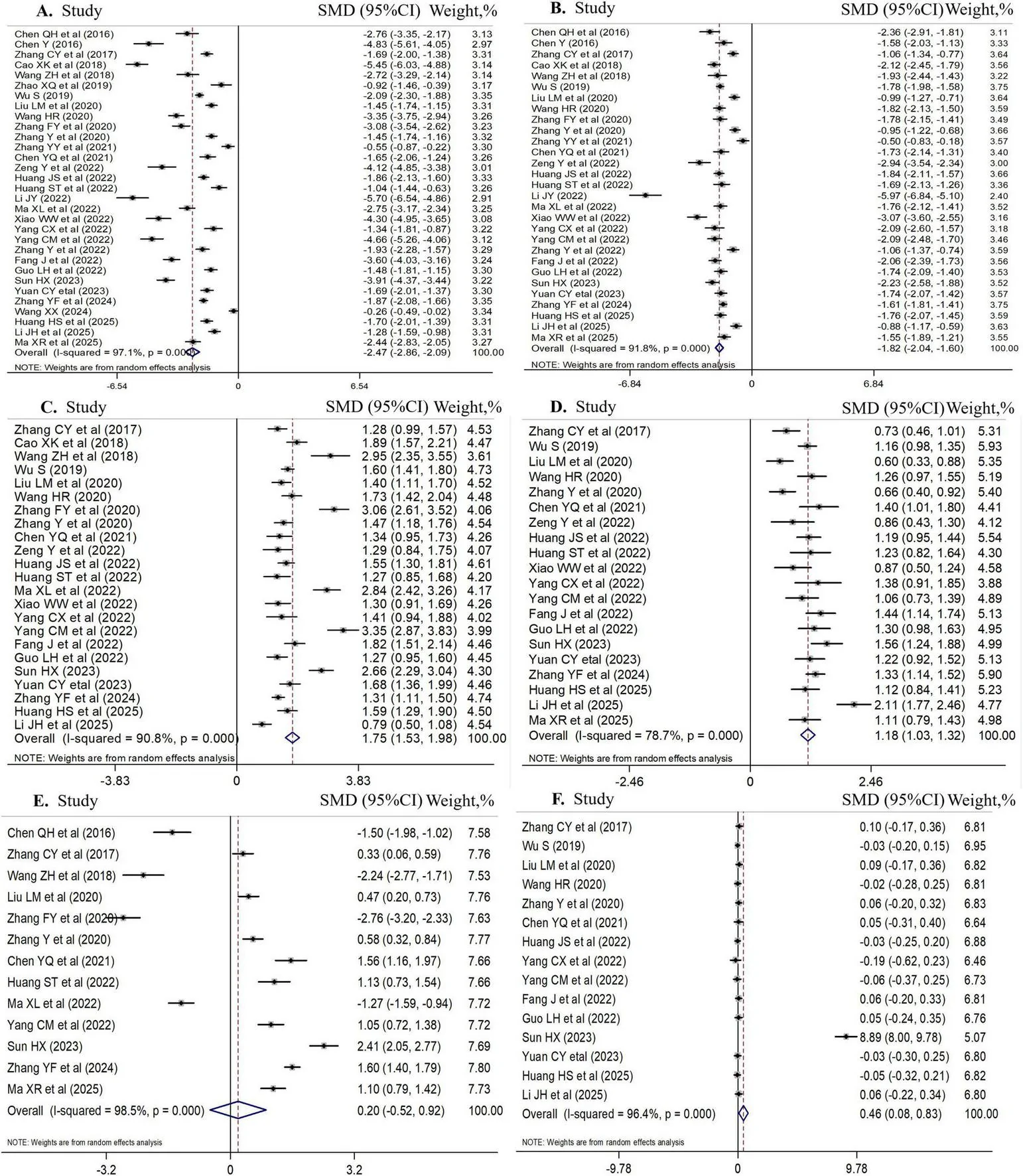
Forest plots of gut microbiota and GDM. **(A)** *Bifidobacterium*. **(B)** *Lactobacillus*. **(C)** *Enterobacteriaceae*. **(D)** *Enterococcus*. **(E)** *Bacteroides*. **(F)** *Fusobacterium*.

### 3.2.2 Lactobacillus and GDM

As illustrated in Figure 2B, the meta-analysis of Lactobacillus included 29 studies (11–15, 17–37, 39–41) comprising 5,813 participants (2,792 GDM cases and 3,021 controls). Significant heterogeneity was observed across studies (I^2^ = 91.80%, *P* < 0.001), necessitating the use of a random-effects model. The pooled analysis revealed a statistically significant reduction in Lactobacillus levels among GDM patients compared to controls (SMD = −1.82, 95%CI: −2.04 to −1.60, *P* < 0.001).

#### 3.2.3 Enterobacteriaceae and GDM

This meta-analysis of Enterobacteriaceae comprised 23 studies (13–15, 17–21, 23–26, 28–31, 33–37, 39, 40) with 2,376 GDM cases and 2,629 controls. Given the substantial heterogeneity across studies (I^2^ = 90.80%, *P* < 0.001), a random-effects model was applied. The pooled estimate revealed significantly elevated levels of Enterobacteriaceae in the GDM group compared to controls (SMD = 1.75, 95%CI: 1.53 to 1.98, *P* < 0.001). As shown in Figure 2C.

#### 3.2.4 Enterococcus and GDM

In the meta-analysis of *Enterococcus* levels and GDM, 20 studies (13, 17–19, 21, 23–26, 29–31, 33–37, 39–41) comprising 4,534 participants (2,138 GDM cases and 2,396 controls) were included. Given the substantial heterogeneity across studies (*I^2^* = 78.70%, *P* < 0.001), a random-effects model was applied. The pooled analysis revealed significantly elevated levels of *Enterococcus* in the GDM group compared to controls (SMD = 1.18, 95% CI: 1.03–1.32, *P* < 0.001). As shown in Figure 2D.

#### 3.2.5 Bacteroides and GDM

As illustrated in Figure 2E, the meta-analysis of *Bacteroides* comprised 13 studies (11, 13, 15, 18, 20, 21, 23, 26, 28, 31, 35, 37, 41) involving 2,491 participants (1,202 GDM cases and 1,289 controls). Given the extreme heterogeneity across studies (*I^2^* = 98.50%, *P* < 0.001), a random-effects model was applied. The pooled estimate revealed no statistically significant difference in *Bacteroides* levels between groups (SMD = 0.20, 95%*CI*: −0.52 to 0.92, *P* = 0.591).

#### 3.2.6 Fusobacterium and GDM

As illustrated in Figure 2F, the meta-analysis of *Fusobacterium* comprised 15 studies (13, 17–19, 21, 23, 25, 30, 31, 33–36, 39, 40) involving 3,517 participants (1,644 GDM cases and 1,873 controls). Due to extreme heterogeneity across studies (*I^2^* = 96.40%, *P* < 0.001), a random-effects model was applied. Under this model, *Fusobacterium* levels were found to be significantly elevated in the GDM group compared to controls (SMD = 0.46, 95% CI: 0.08–0.83, *P* = 0.018).

### 3.3 Analysis of publication bias

Publication bias was assessed for the target gut microbiota (each comprising > 10 included studies) using funnel plots supplemented by Begg’s test. The funnel plots for *Enterococcus*, *Bacteroides*, and *Fusobacterium* exhibited relative symmetry, and Begg’s test indicated no statistically significant publication bias for these associations (all *P* > 0.05). In contrast, the funnel plots for *Bifidobacterium*, *Lactobacillus*, and *Enterobacteriaceae* displayed asymmetry, and Begg’s test suggested evidence of significant publication bias in the meta-analyses of their relationships with GDM (all *P* < 0.05). See Figure 3.

**FIGURE 3:**
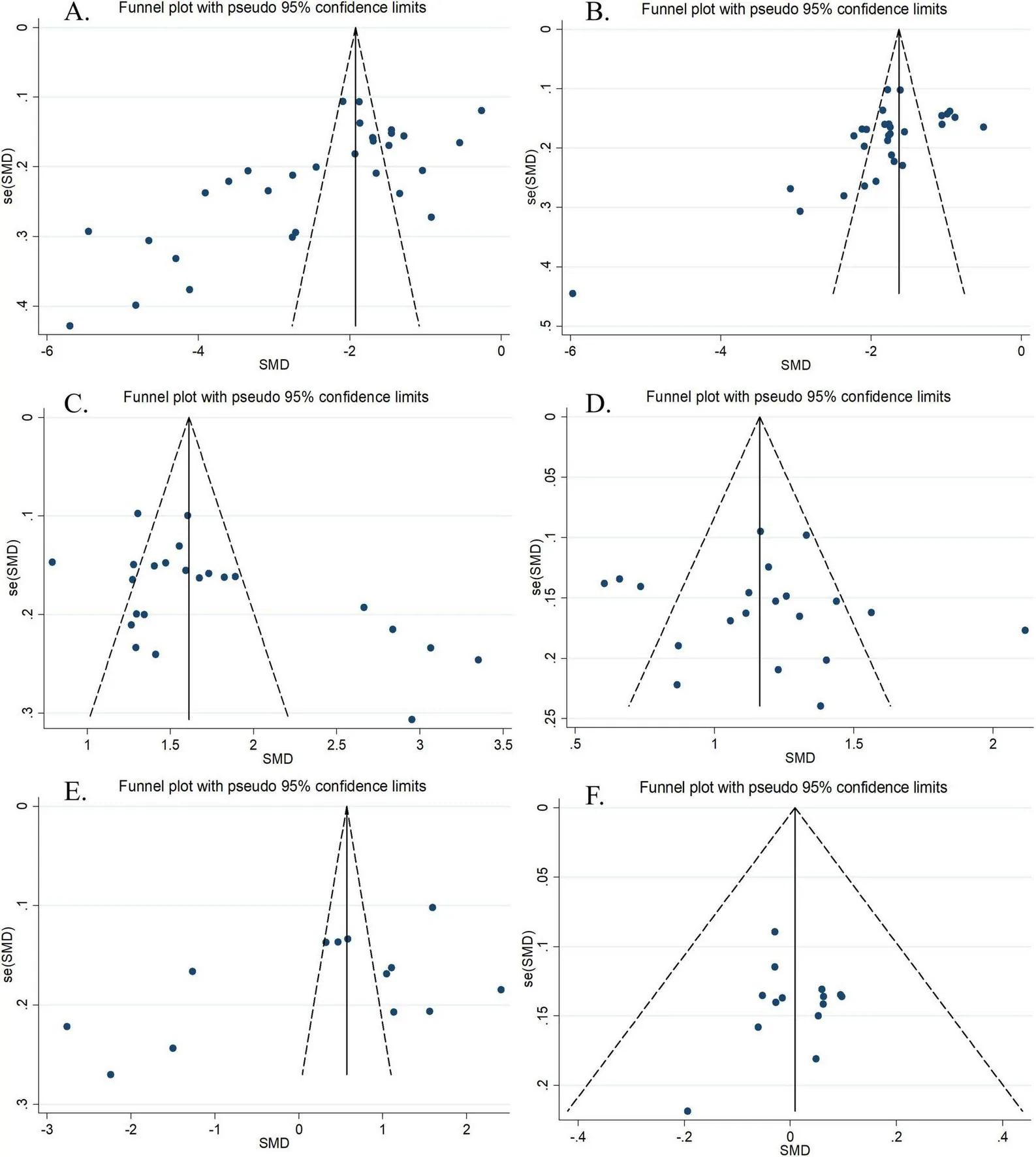
Funnel plots for publication bias. **(A)** *Bifidobacterium*. **(B)** *Lactobacillus*. **(C)** *Enterobacteriaceae*. **(D)** *Enterococcus*. **(E)** *Bacteroides*. **(F)** *Fusobacterium*.

To assess how the detected publication bias affected the robustness of the pooled estimates, trim-and-fill analyses were conducted under a random-effects model using the linear trimming method. The procedure imputed 2, 9, and 0 hypothetically missing studies for *Bifidobacterium*, *Lactobacillus*, and *Enterobacteriaceae*, respectively. The bias-corrected random-effects SMDs were −2.632 (95% CI: −3.104 to −2.160) for *Bifidobacterium*, −2.158 (95% CI: −2.435 to −1.882) for *Lactobacillus*, and 1.753 (95% CI: 1.531 to 1.975) for *Enterobacteriaceae*. Although the results showed some evidence of publication bias for certain outcomes, the bias-corrected estimates remained statistically significant and directionally consistent with the original findings, suggesting that the main conclusions were unlikely to be substantially affected by potential publication bias. See Supplementary Table 1.

### 3.4 Sensitivity analysis

Sensitivity analyses were performed using the leave-one-out method to evaluate the robustness of the pooled effect sizes for the associations between target gut microbiota and GDM. For *Bifidobacterium*, *Lactobacillus*, *Enterobacteriaceae*, *Enterococcus*, and *Bacteroides*, the pooled estimates and their 95% CIs remained substantially unchanged upon sequential omission of individual studies, with no reversal of conclusions, indicating highly robust associations In contrast, for *Fusobacterium*, the removal of an extreme outlier (35) yielded consistent findings across the remaining studies, confirming the stability of the association. For Bifidobacterium, “leave-one-out” sensitivity analysis identified Li (27) and Cao et al. (14) as potential outliers with exceptionally large effect sizes. After excluding these two studies, the pooled SMD was −2.27 (95% CI: −2.62 to −1.91), which remained statistically significant and directionally consistent with the original estimate (SMD = −2.47, 95% CI: −2.86 to −2.09). For Lactobacillus, exclusion of Li (27) yielded a recalculated SMD of −1.71 (95% CI: −1.90 to −1.52), compared with the original estimate of −1.82 (95% CI: −2.04 to −1.60), which remained statistically significant and directionally unchanged. These results confirm that the overall pooled effects for both genera are robust and not disproportionately driven by extreme individual studies. See Figure 4

**FIGURE 4:**
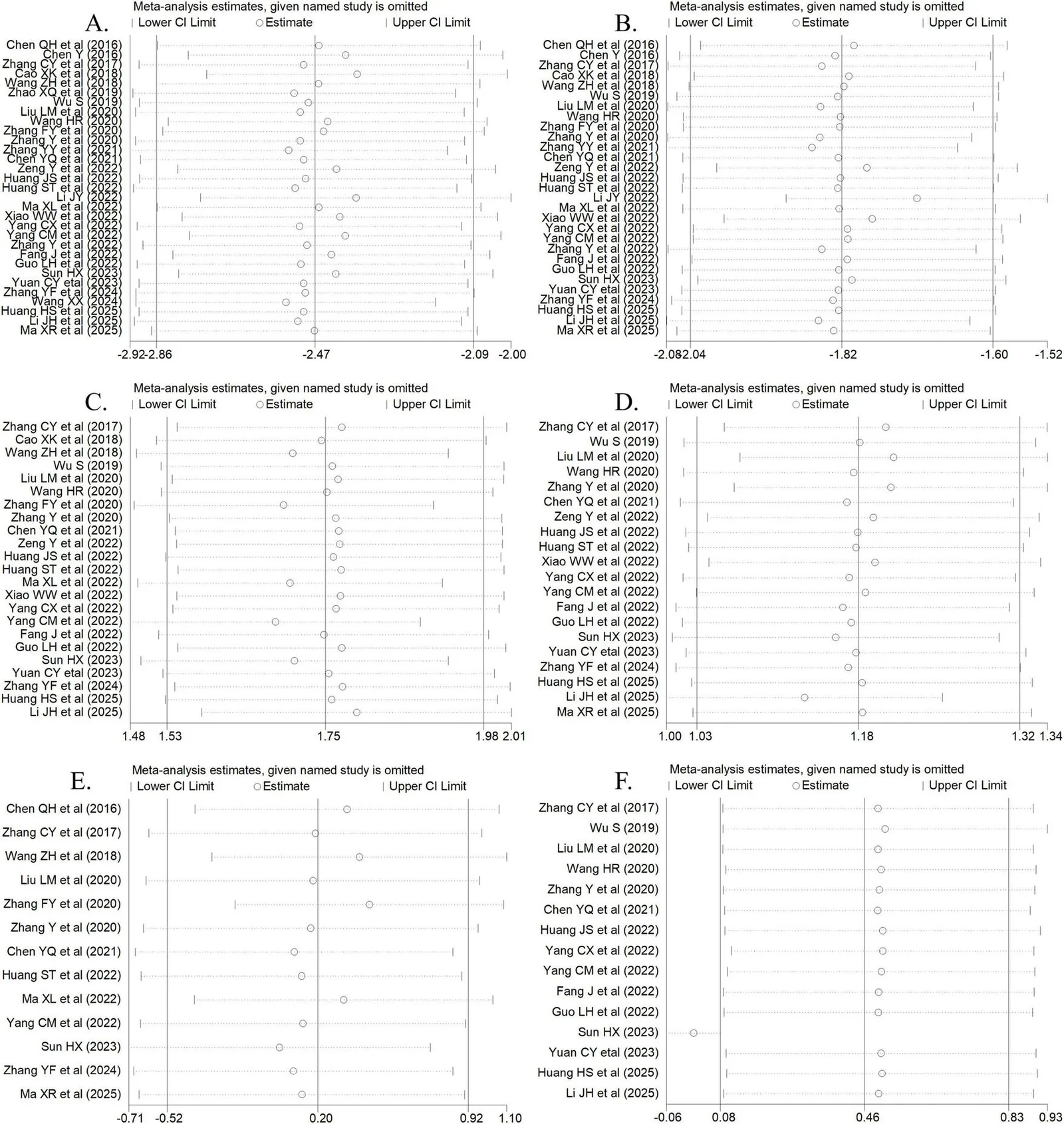
Leave-one-out sensitivity analyses. **(A)** *Bifidobacterium*. **(B)** *Lactobacillus*. **(C)** *Enterobacteriaceae*. **(D)** *Enterococcus*. **(E)** *Bacteroides*. **(F)** *Fusobacterium*.

### 3.5 Heterogeneity source analysis

Given the substantial heterogeneity observed in the meta-analyses of the associations between target gut microbiota and GDM, we performed meta-regression analyses to explore potential sources of heterogeneity. Covariates including publication year, geographical region, detection method, pre-pregnancy BMI (pre-BMI), gestational week, and parity were incorporated into the models to quantify their contribution to the observed variance. Meta-regression analysis revealed that publication year was a significant moderator explaining the heterogeneity observed in studies of *Enterococcus* (*P* = 0.012) and *Bacteroides* (*P* = 0.035). Additionally, geographical region was identified as a primary source of heterogeneity for *Fusobacterium* (*P* = 0.043). Notably, detection method also emerged as a significant contributor to the heterogeneity in studies of both *Enterococcus* (*P* = 0.025) and *Fusobacterium* (*P* = 0.044). See Table 3.

**TABLE 3:** Meta-regression explores the sources of significant heterogeneity in data.

Gut microbiota	Meta-regression (t / *P*)					
	Year	Region	Method	pre-BMI	Gestational week	Gravidity
*Bifidobacterium*	1.31/0.201	−0.97/0.339	−0.21/0.831	−0.12/0.902	−1.34/0.191	−0.26/0.793
*Lactobacillus*	−0.21/0.835	−1.12/0.273	−0.76/0.454	0.11/0.916	−1.42/0.167	0.06/0.954
*Enterobacteriaceae*	−0.90/0.376	0.73/0.476	0.73/0.471	0.60/0.722	−0.36/0.167	1.40/0.175
*Enterococcus*	2.78/0.012	−0.23/0.819	2.45/0.025	0.14/0.891	1.46/0.162	−1.26/0.225
*Bacteroides*	2.40/0.035	1.21/0.252	1.05/0.315	0.33/0.746	2.11/0.059	−0.49/0.635
*Fusobacterium*	0.65/0.528	2.24/0.043	2.23/0.044	0.60/0.559	1.01/0.333	0.99/0.340

Additionally, the leave-one-out analysis revealed that after excluding the study by Sun et al. (35), the heterogeneity among the pooled data for *Fusobacterium* dropped to 0% (*P* = 0.998), suggesting that this study was likely a major source of the significant heterogeneity observed in the *Fusobacterium* data.

## 4 Discussion

The human gut harbors a complex ecosystem comprising approximately 500 to 1,000 bacterial species, with the phyla *Firmicutes* and *Bacteroidetes* constituting the vast majority. Within this community, beneficial genera such as *Bifidobacterium* and *Lactobacillus* play pivotal roles in preserving microbial homeostasis (42, 43). Accumulating evidence indicates that the gut microbiota orchestrates a range of physiological processes, including digestion, nutrient absorption, energy metabolism, and immune modulation, while simultaneously reinforcing the intestinal barrier (44, 45). Dysregulation characterized by structural damage can lead to metabolic endotoxemia and chronic low-grade inflammation, thereby driving the pathogenesis and progression of metabolic disorders such as gestational diabetes mellitus (GDM) (46).

Consistent with prior domestic and international meta-analyses (47, 48), this study demonstrated significantly lower levels of the beneficial genera *Bifidobacterium* and *Lactobacillus*in GDM patients, alongside elevated abundances of opportunistic pathogens from the family *Enterobacteriaceae* and the genus *Enterococcus*. Two primary mechanisms underpin this association. Firstly, commensal bacteria such as *Bifidobacterium* and *Lactobacillus* maintain gut barrier integrity and metabolic homeostasis by fermenting dietary fiber into short-chain fatty acids (SCFAs). SCFAs serve as energy substrates for colonocytes, reinforce mucosal integrity, and stimulate glucagon-like peptide-1 (GLP-1) release via G-protein-coupled receptor activation, thereby enhancing insulin sensitivity (49, 50). Conversely, depletion of these probiotics reduces SCFA production, potentially increasing intestinal permeability and triggering metabolic endotoxemia, which exacerbates insulin resistance (51). Secondly, gut dysbiosis facilitates the translocation of lipopolysaccharide (LPS), a key component of the *Enterobacteriaceae* cell wall entering the circulation. This elicits chronic low-grade inflammation by upregulating pro-inflammatory cytokines (such as TNF-α and IL-6), which interferes with insulin signaling and drives GDM pathogenesis (52, 53).

No significant overall association between *Bacteroides* and GDM risk was observed. However, the forest plot revealed substantial inter-study inconsistency. This variability likely reflects the functional duality of *Bacteroides*. As proposed by Zafar et al. (54), these microbes can exert probiotic effects via SCFA-mediated barrier maintenance, yet may drive inflammation through LPS translocation when the barrier is compromised. Expanding on this, Fansler et al. (55) introduced the “niche remodeling” theory, positing that *Bacteroides* adaptively shifts from reinforcing the mucus layer (via propionate production under high-fiber conditions) to degrading it and releasing LPS (under fiber deficiency or dysbiosis), thereby transitioning from “friend” to “foe.” Consequently, the inconsistent findings underscore how dietary patterns, specific subspecies, and the gut microenvironment critically dictate the metabolic role of *Bacteroides*.

This study has several limitations. First, the exclusive inclusion of case-control studies precludes causal inference; thus, high-quality prospective cohort studies are warranted to establish the temporality between gut dysbiosis and GDM onset. Second, the generalizability of our findings to other Asian populations or to geographically and ethnically diverse subpopulations within China may be limited by regional variations in diet, lifestyle, and host genetics; researchers attempting replication in other settings should prioritize region-specific validation and standardized protocols. Third, substantial heterogeneity persisted in several meta-analyses. Subgroup analyses implicated detection methodologies and gestational age as potential sources, while funnel plot asymmetry and Begg’s test results for *Bifidobacterium*, *Lactobacillus*, and *Enterobacteriaceae* suggested non-trivial publication bias, likely driven by the under-reporting of negative findings. This underscores the current lack of standardization in GDM microbiome research regarding population characteristics (such as geography, diet, BMI), sample processing, and sequencing platforms. Finally, our analysis was confined to the genus level, limiting insights into species-specific or functional genomic alterations. Future multi-omics approaches integrating metagenomics and metabolomics are essential to fully elucidate the role of microbial dysfunction in GDM pathogenesis.

Notwithstanding its limitations, this study possesses considerable clinical relevance. By providing large-sample, evidence-based insights into the gut microbiota profile of Chinese GDM patients, it identifies a defining dysbiotic signature: that is a decline in keystone genera (*Bifidobacterium* and *Lactobacillus*) coupled with an expansion of opportunistic pathogens (*Enterobacteriaceae* and *Enterococcus*). These findings suggest that microbiota modulation via probiotics, prebiotics, or dietary modification, may constitute an effective adjunctive strategy for GDM management. This premise is supported by existing meta-analyses confirming that probiotic supplementation significantly ameliorates fasting glucose, insulin levels, and HOMA-IR in GDM patients.

In conclusion, Chinese GDM patients exhibit a distinct dysbiotic profile characterized by depleted beneficial genera (*Bifidobacterium*, *Lactobacillus*) and enriched opportunistic pathogens (*Enterobacteriaceae*, *Enterococcus*). These taxonomic shifts represent key microbiological hallmarks in GDM pathogenesis. Furthermore, personalized microbiome-based interventions, tailored to regional dietary patterns and host metabolic phenotypes, are essential to advance early warning systems and precision prevention strategies for GDM.

## Data Availability

The original contributions presented in this study are included in this article/Supplementary material, further inquiries can be directed to the corresponding authors.
