# Supplementary material for: A systematic analysis of gut microbiome characteristics in Chinese patients with gestational diabetes mellitus
Source: Front Nutr. 2026 Sep 22;13:1902356. doi: 10.3389/fnut.2026.1902356 (PMC13638395; doi:10.3389/fnut.2026.1902356)
Supplement: Supplementary file 1 [file Table_1.docx]

**Supplementary Table 1 Sensitivity analysis for publication bias: trim-and-fill adjusted pooled effect sizes forBifidobacterium,Lactobacillus, and Enterobacteriaceae**

| Taxon | Original  SMD (95% CI) | Imputed missing studies (n) | Bias-corrected  SMD (95% CI) | Directional consistency |
| --- | --- | --- | --- | --- |
| *Bifidobacterium* | -2.474 (-2.859, -2.089) | 2 | -2.632 (-3.104, -2.160) | Yes |
| *Lactobacillus* | -1.822 (-2.041, -1.603) | 9 | -2.158 (-2.435, -1.882) | Yes |
| *Enterobacteriaceae* | 1.753 (1.531, 1.975) | 0 | 1.753 (1.531, 1.975) | Yes |

Note: Trim-and-fill analyses were performed using the linear trimming method under a random-effects model. SMD: standardized mean difference; CI: confidence interval. Directional consistency indicates that the bias-corrected effect estimate remained in the same direction and remained statistically significant (all *P* < 0.05) as the original estimate.
